# Polymeric Hydrogels as Technology Platform for Drug Delivery Applications

**DOI:** 10.3390/gels3030025

**Published:** 2017-07-03

**Authors:** Alejandro Sosnik, Katia P. Seremeta

**Affiliations:** 1 Laboratory of Pharmaceutical Nanomaterials Science, Department of Materials Science and Engineering, Technion-Israel Institute of Technology, Technion City, Haifa, 3200003, Israel; 2 Departamento de Ciencias Básicas y Aplicadas, Universidad Nacional del Chaco Austral, Pcia. Roque Sáenz Peña, Chaco, 3700, Argentina; kseremeta@uncaus.edu.ar; 3 Consejo Nacional de Investigaciones Científicas y Técnicas (CONICET), Pcia. Roque Sáenz Peña, 3700, Argentina

**Keywords:** hydrogels, drug delivery, bioadhesion and mucoadhesion, oral delivery, topical delivery, vaginal delivery, rectal delivery, ocular delivery, transdermal delivery

## Abstract

Hydrogels have become key players in the field of drug delivery owing to their great versatility in terms of composition and adjustability to various administration routes, from parenteral (e.g., intravenous) to non-parenteral (e.g., oral, topical) ones. In addition, based on the envisioned application, the design of bioadhesive or mucoadhesive hydrogels with prolonged residence time in the administration site may be beneficial. For example, hydrogels are used as wound dressings and patches for local and systemic therapy. In a similar way, they can be applied in the vaginal tract for local treatment or in the nasal cavity for a similar goal or, conversely, to target the central nervous system by the nose-to-brain pathway. Overall, hydrogels have demonstrated outstanding capabilities to ensure patient compliance, while achieving long-term therapeutic effects. The present work overviews the most relevant and recent applications of hydrogels in drug delivery with special emphasis on mucosal routes.

## 1. Introduction

Hydrogels are hydrophilic viscoelastic materials formed by a polymeric network with physical and/or chemical crosslinks that absorb and retain large amounts of water (several times their dry weight) and swell, while maintaining their 3D structure, mechanical strength and elasticity [1,2,3,4,5]. In addition, hydrogels can be easily modified with functional groups and display pores with well-defined sizes that can be controlled by the crosslinking density and that can be sensitive to external stimuli such as pH, temperature and magnetic field [6]. In a hydrogel, polymer chains create a characteristic 3D matrix with interstitial spaces that are capable of harboring aqueous fluids such as the physiological ones [4]. Moreover, the high water content provides an environment for the diffusion of oxygen, nutrients and other small molecules that are critical for cell growth and proliferation [7]. Thus, molecules of different drugs can be incorporated into the hydrogel and diffuse from the interstitial spaces to the biological medium, allowing their use as a reservoir for controlled release applications [1]. Therefore, over the years, hydrogels became key players in the biomedical field in general and pharmaceutical research and development in particular, employing different invasive and minimally invasive administration routes [8]. Peppas et al. [9] have done an enormous contribution to the understanding of the mechanisms involved in the release from different systems, from moderately or poorly swellable [10] to semi-crystalline hydrogels [11].

The choice of the material for the synthesis of the hydrogel depends on the application and ranges from fully synthetic polymers such as poly(ethylene glycol) (PEG) and poly(vinyl alcohol) (PVA) to natural ones such as polysaccharides (e.g., hyaluronic acid, alginate, chitosan) and proteins (e.g., gelatin) [12,13]. For example, the use of chitosan hydrogel scaffolds for controlled and localized delivery of drugs that promote wound healing was recently reviewed by Elviri et al. [14]. 

**Figure 1:**
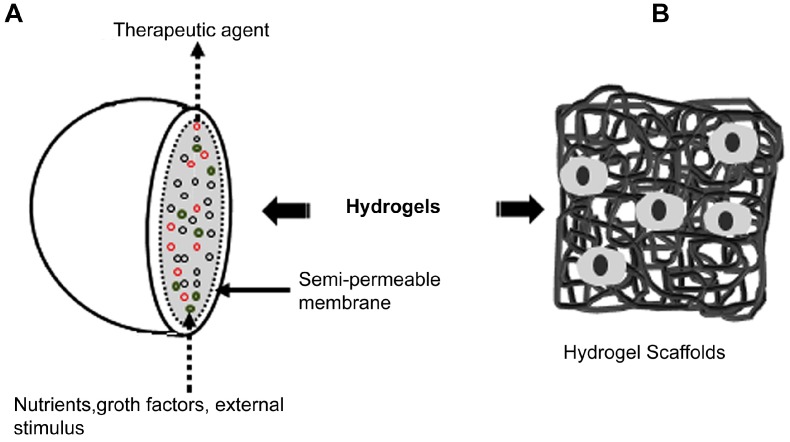
Hydrogels and tissue engineering. Schematic diagram of the use of hydrogels in (**A**) microencapsulation and (**B**) tissue-engineering scaffold. (Reprinted with permission from reference [16]. Copyright 2014 Elsevier).

Hydrogels can be prepared using different methods and precursors that allow the adjustment of properties such as pore size, mechanical strength, degradability and rate of degradation [13,15]. For example, Silva et al. [16] reviewed the manufacture and use of hydrogels based on fibrous proteins (e.g., silk, keratins, elastin, resilins), which are one of the most versatile biomaterials for tissue engineering. These scaffolds can be also used as carriers for the localized delivery of growth factors, enzymes, and drugs (Figure 1).

As explained above, properties of hydrogels such as pore size may be controlled by the production conditions and/or the modification of the polymers or the weight ratio between them. For example, Zhang et al. [17] studied the properties of PEG diacrylate (PEGDA) hydrogels by varying the molecular weight of the precursor (400–2000 g/mol) or the ratio between them (100/0, 40/20, 30/30, 20/40, 0/100). Systems were photo-polymerized under visible light at a total polymer concentration of 60% w/w. Results showed that properties such as swelling, wetting, elastic modulus, transparency and microstructure changed when modifying these synthesis parameters.

An important characteristic of hydrogels is their similarity with the extracellular matrix, thus allowing their use as scaffolds for tissue engineering and regenerative medicine [4]. Slaughter et al. [18] extensively reviewed the application of hydrogels in regenerative medicine.

Overall, hydrogels have emerged as an extremely versatile technology platform for tissue engineering and drug delivery. In this work, we will overview the development and use of polymeric hydrogels for advanced drug delivery systems. Even though the literature is very profuse, we will focus mainly on most recent works that introduce new concepts and methods for their synthesis, characterization and/or administration. In this context, the field of tissue engineering is beyond the scope of the review. At the same time, it is worth stressing that aiming to improve tissue regeneration and repair, some researchers used hydrogel scaffolds for the localized delivery of a variety of active compounds, from drugs to biologicals. Moreover, the use of some innovative hydrogels conceived for tissue engineering could be extended to other biomedical applications, including drug delivery. Thus, some examples at the interface of these fields, namely drug delivery and tissue engineering, will be also briefly discussed. 

## 2. Polymeric Hydrogels for Drug Delivery Applications

An interpenetrating polymer network is a 3D network composed of two or more networks, where the polymer chains are partially or fully intertwined at a molecular level, but not covalently linked to each other and thus, they cannot be separated unless chemical bonds are broken. In this sense, Naseri et al. [19] designed hydrogels of a double cross-linked interpenetrating polymer network based on cellulose nanocrystals in a matrix of sodium alginate and gelatin by freeze-drying. A homogeneous solution of sodium alginate and gelatin was mixed with a suspension of nanocrystalline cellulose and allowed to dry for three days. This dry blend was put in contact with divalent calcium ions (Ca^2+^) and then with genipin, a natural aglycone derived from the fruit of *Gardenia jasminoides*; the former crosslinks alginate, while the latter gelatin. Finally, samples were lyophilized for 24 h to obtain sponges for potential use as a substitute for cartilage. Cellulose nanocrystals have garnered much interest in biomedical applications due to have showed improve the dimensional stability and the mechanical strength of hydrogels and facilitate the release of drugs.

**Figure 2:**
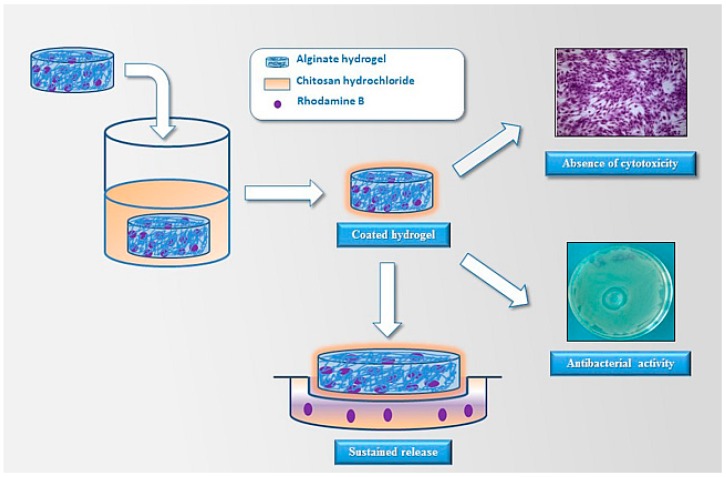
Preparation of alginate hydrogels coated with chitosan for wound dressing. (Reprinted from reference [20]).

Hydrogels formed from self-assembled biopolymer networks are particularly interesting in better mimicking the microenvironment of native tissues as they show structural, chemical and mechanical similarities to the extracellular matrix and good biological compatibility. Tissue engineering scaffolds play a fundamental role in providing a native environment that mimics tissues for the cells, with the aim of promoting regeneration of the desired tissue [16]. One of the main applications of hydrogels is the treatment of wounds, an application that usually demands the use of biomaterials that promote tissue repair or regeneration and prevent infections. In this context, Straccia et al. [20] developed alginate hydrogels coated with chitosan as wound dressing. The coating was used to confer antibacterial activity and to delay the release of charged hydrophilic drugs into the alginate (Figure 2). 

**Figure 3:**
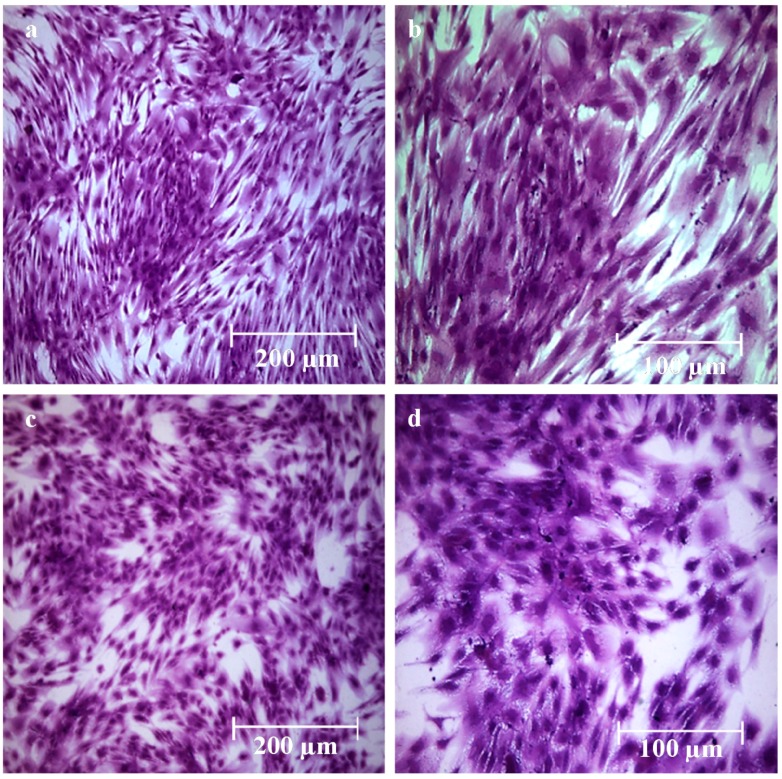
(**a**,**b**) Microscopic images of mesenchymal stromal cells (MSC) cultured for seven days in control culture medium after crystal violet staining; (**c**,**d**) microscopic images of MSC cultured for seven days in 0.1% chitosan hydrochloride culture medium after crystal violet staining. (Reprinted from reference [20]).

All the coated hydrogels retained their transparency, a desirable feature in wound dressings that allows visual inspection of the lesion bed throughout the healing process. In vitro assays with human mesenchymal stromal cells (MSC) confirmed the absence of acute toxicity of the coating biomaterial (Figure 3) [20].

The release characteristics were assayed using rhodamine B as the hydrophilic model drug and compared with that of the uncoated counterparts. The presence of the chitosan coating served as a barrier that delayed the diffusion of rhodamine B and, therefore, resulted in a decrease of the release kinetics. Finally, the antibacterial activity of coated hydrogels against *Escherichia coli* was demonstrated. 

Currently, new biomaterials modified with cyclodextrins (CDs) are being investigated as advanced delivery systems for both hydrophilic and hydrophobic drugs [21]. CDs are natural cyclic oligosaccharides consisting of α-d-glucopyranose units joined by α-1,4-type bonds and provided with a central lipophilic cavity and a hydrophilic outer surface that allow the formation of inclusion or non-inclusion complexes, thus improving the physicochemical properties of drugs, in particular, those displaying low aqueous solubility. This ability can be imparted to polymer networks in which the CDs are chemically grafted or cross-linked [22]. The combination of CDs and hydrogels in one single biomaterial leads to synergistic properties: the hydrophilic polymer network improves the biocompatibility and prevents dilution in the physiological environment, increasing the stability of the complexes, while CDs provide a more controlled release of the active cargo [7]. For example, Mennini et al. [23] developed CD-containing ethyleneglycol diglycidyl ether hydrogels for vaginal administration of dehydroepiandrosterone in the treatment of postmenopausal symptoms. Ovules combined the controlled release and mucoadhesion properties of the hydrogel, and the dissolution capacity of the CD to overcome its low bioavailability associated with poor aqueous solubility. 

## 3. Preformed Hydrogels versus Hydrogels Formed Upon Implantation (In Situ)

**Figure 4:**
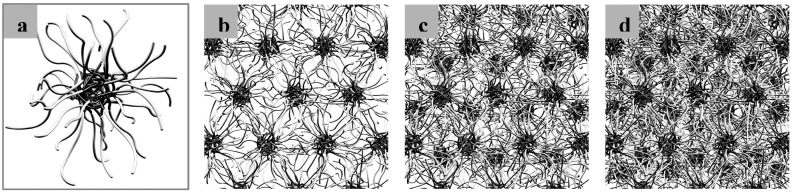
Schematic representations of Pluronic^®^ F127 micelles: (**a**) single micelle with spherical core-shell geometry; (**b**) single 2D hexagonally packed layer of micelles; (**c**) two 2D hexagonally packed layers of micelles (AB); and (**d**) three layers with ABC (or Faced Centered Cubic, FCC) stacking sequence structure. (**b**–**d**) correspond to the radial geometry. (Reprinted with permission from reference [32]. Copyright 2007 American Chemical Society).

In early years, most hydrogels intended for drug delivery were produced prior to administration (known as preformed hydrogels) and depending on their size (macroscopic or microscopic), they were administered by invasive or minimally invasive procedures. In other words, preformed hydrogels are simple viscous solutions or films, which gel outside the body and do not undergo a modification of their properties (e.g., rheological or mechanical) after administration. More recently, the development of systems that are deployable in the body as liquids or semi-solids and undergo a transition to solidify in situ emerged as a very appealing strategy to ensure patient compliance and comfort [24]. Different mechanisms can be exploited to achieve the transition, from in situ polymerization by photo-initiated reactions to the use of stimuli-responsive polymers that change their properties in response to changes in temperature, pH, and ionic strength, under physiological conditions. In addition, hydrogels may respond to externally applied stimuli such as ultrasound, electromagnetic field or light that may be employed to release the cargo with modified kinetics (e.g., pulsatile release). In this context, Tsitsilianis [25] reviewed stimuli-responsive reversible hydrogels with tunable gel properties, such as injectability and responsiveness, mesh size, mechanical strength and dynamics. These hydrogels provide promising platforms for the encapsulation and delivery of drugs and cells in a variety of biomedical applications [1,26,27,28]. For example, one of the most widely exploited polymers for the synthesis of in situ formed hydrogels used in the delivery of drugs are poly(ethylene oxide)-*b*-poly(propylene oxide) (PEO-PPO) block copolymers known as poloxamers or Pluronic^®^ (linear and bifunctional) and poloxamines or Tetronic^®^ (branched and tetrafunctional) [29]. Due to their amphiphilic nature, these copolymers can self-assemble and form spherical polymeric micelles in aqueous solution above the critical micellar concentration (CMC). In addition, above the critical concentration and temperature of gelation, the self-assembled polymeric micelles are tightly bound to produce a physically cross-linked hydrogel structure [30,31,32]. Figure 4 shows the structure of polymeric micelles made of Pluronic^®^ copolymers and their gelation once the concentration is increased [32].

**Figure 5:**
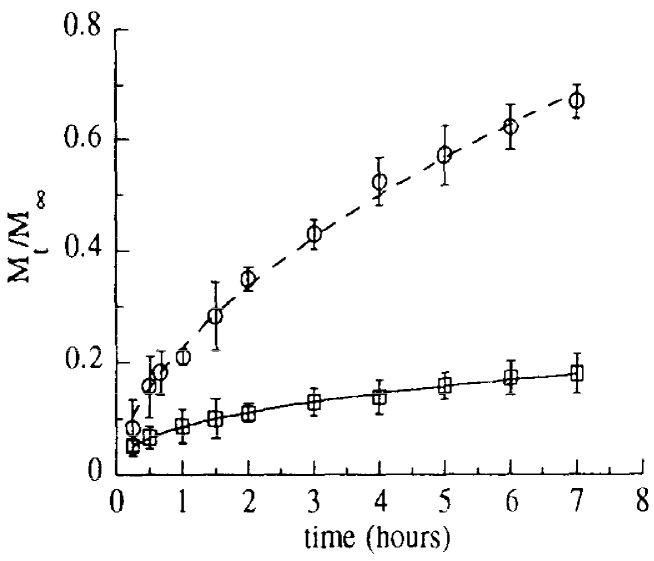
Tetracycline release profiles from poloxamer (- - -) and monoglyceride (―) based gels. Kinetics were determined by equilibrium dialysis. The reported values represent the average of five independent experiments, bars = S.D. (Reprinted with permission from reference [34]. Copyright 1996 Elsevier).

Thus, these systems exhibit sol-gel transition properties in response to temperature in aqueous solution usually around the physiological temperature and, therefore, they can be administered as liquids at room temperature and undergo gelation at body temperature. In addition, the gelation temperature of these formulations decreases with a concentration increase [31]. It is worth stressing that high copolymer concentration is usually required to form the gel. For example, 25% *w*/*v* poloxamers are required to form a rigid gel after ocular instillation [33]. Moreover, regardless of the concentration, the micro-viscosity properties are relatively poor, which causes fast dilution in the biological fluids and release of the payload (Figure 5) [34] and eventually elimination from the administration site, especially in those with significant biological fluid flow (e.g., mouth, vagina). 

Aiming to reduce the copolymer content and improve the gelation properties, these gel-forming biomaterials were modified [35,36]. For example, Cohn et al. [37] polymerized poloxamer precursors and increased their molecular weight, resulting in a dramatic improvement of the rheological properties and the physical stability in aqueous media. In addition, it was found that a reduction in the concentration of the gel-forming copolymer could be achieved by the incorporation of a suitable viscosity-enhancing polymer [38].

### 3.1. Preformed Hydrogels

As mentioned above, preformed hydrogels constitute the first generation and have been extensively explored in biomedical applications in general, and drug release in particular. This review does not attempt to be a comprehensive compendium of all the literature on the subject and, therefore, some recent works of especial interest are briefly described. Huang et al. [39] obtained a hybrid hydrogel membrane composed of carboxymethylcellulose (CMC) enriched with berberine (anti-inflammatory and antimicrobial agent) and hyaluronic acid to be used as an anti-adhesive barrier and drug delivery system with excellent anti-inflammatory and tissue regeneration properties to prevent post-surgical adhesions. PVA, the surfactant Tween^®^ 80 and a natural oil were added as membrane-forming agent, emulsifier and lubricant, respectively. The homogenous mixture was placed in a mold and subjected to heating at 37 °C for 3 h to form a dry membrane. A hydrogel of CMC (loaded with berberine) and hyaluronic acid in a ratio of 30:70 presented optimal plasticity and inhibited 60% of lipopolysaccharide-stimulated inflammation in RAW264.7 macrophages in vitro.

Preformed hydrogels can also be injected as solids [40]. In this case, the solid system can be “thinned” by applying a sufficiently high shear stress and thus, reduce its viscosity. Once at the implantation site and the stress being eliminated, the material immediately recovers the original mechanical properties of the solid. Thus, these drug delivery systems become a more benign treatment when compared to conventional surgery that involves tissue damage and later suture. Due to this property, shear thinning and thixotropic systems are often included in the category of injectable biomaterials [40]. Recently, Riber et al. [41] developed a new technology as a drug delivery platform consisting of a network of interpenetrating silicone hydrogel polymers impregnated with a broad-spectrum antimicrobial compound such as irgasan, to eliminate plasmids. The spread of antimicrobial resistance, generally mediated by the horizontal transfer of plasmids, limits treatment options for bacterial infections. Exposure of bacteria (e.g., *Escherichia coli K-12*) to this interpenetrating network for 24 h resulted in a significant loss of plasmid (2.8–4.7%) (*p* < 0.05). To further increase the plasmid loss, the concentration of impregnated irgasan should be increased, or conversely, a constant exposure ensured over time. Both options would likely result in severely damaged cell growth conditions and the formation of an adaptive response leading to the development of drug resistance, suggesting the limitations of this compound in future therapeutic and medical applications. However, as a drug delivery device, the hydrogel portion of the matrix network extends the range of drugs that can be transported, stored and subsequently released, including both hydrophilic molecules such as silver ion complexes, sulfonamides and antimicrobial peptides as hydrophobic compounds.

Hydrogel-based contact lenses can also be designed using suitable polymers. For example, Tummala et al. [42] developed a highly transparent hydrogel with high water content (>90%) by combining PVA with nanocellulose (NC) to obtain model contact lenses without compromising their transparency. NC was used to strengthen the PVA hydrogel and facilitate the manipulation of the lenses. Hydrogels showed a refractive index close to that of pure water, high transparency to visible light and very good blocking properties to ultraviolet radiation. In addition, they were rigid and flexible enough to adapt well to a convex surface such as the eye and exhibit high elasticity without being ruptured under stretching. The high water content can favor optical transparency, give good comfort of use, high oxygen permeability, low protein deposition and high biocompatibility. The hygroscopic nature of NC could also contribute to the retention of water over time, a fundamental property for a medical device that is exposed to air. With the introduction of additional molecular features (e.g., modification with CDs), this work appears as a platform of value for the development of medicated contact lenses [43].

### 3.2. Hydrogels Formed In Situ

**Figure 6:**
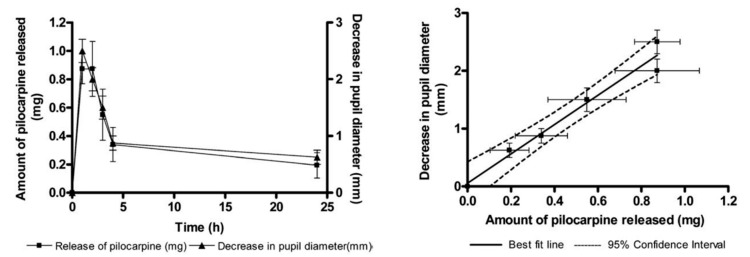
Correlation between in vitro pilocarpine release and pupillary constriction obtained in vivo. A linear correlation is evident with an *R*^2^ of 0.97. As the amount of pilocarpine available for absorption decreases, a corresponding increase in pupil diameter is observed. Data are reported as mean ± SEM. Solid line indicates the best-fit line and dashed line indicates the 95% confidence interval. (Reprinted with permission from reference [1]. Copyright 2009 Elsevier).

As discussed above, in situ generated hydrogels are liquid at the administration time and display a transition due to structural changes induced by external stimuli or in the biological environment [44]. This approach is very advantageous, for example, in ocular delivery as these vehicles can be easily applied as a liquid that ensures complete coverage of the eye being stabilized in situ due to gelation, in contrast to pre-gelled formulations that show lower adjustability [1]. In addition, in a minimally invasive manner, a liquid can be injected for in situ formation of a matrix in the development of injectable intraocular lenses [17]. Following this conceptual approach, Anumolu et al. [1] developed fast crosslinking thiolated PEG hydrogels that are stabilized at physiological pH via S-S bonds. Then, these stable viscoelastic hydrogels were loaded with pilocarpine for controlled ocular delivery and subsequent pupillary constriction. In vivo studies in rabbits showed that the hydrogels ensured pupillary constriction for 24 h after administration, eight times more than an aqueous solution of the same drug (Figure 6).

**Figure 7:**
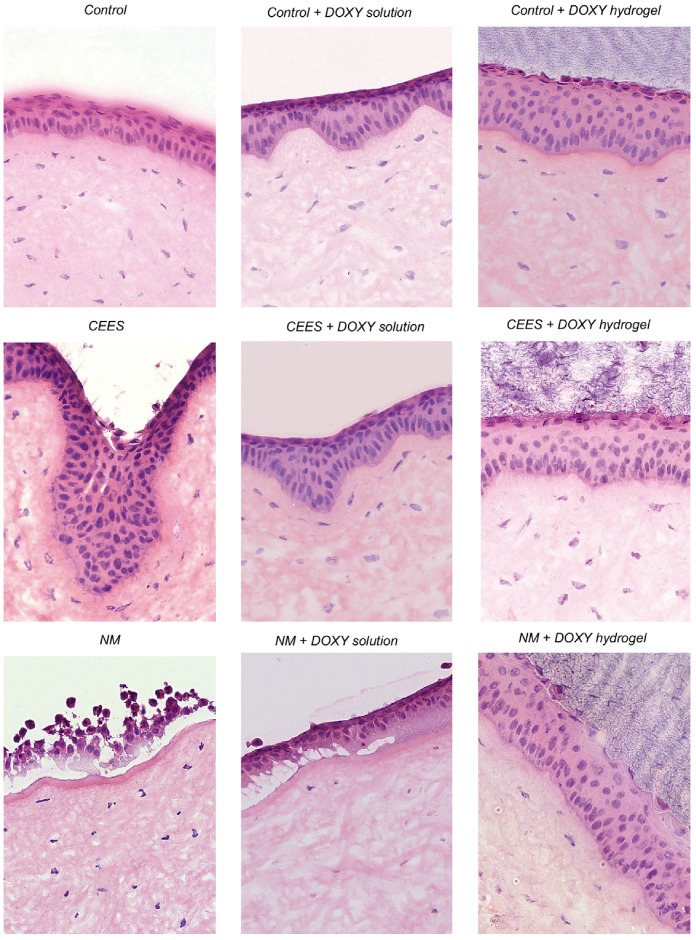
Hematoxylin and Eosin staining to visualize the histology of CEES and NM-exposed corneas treated for 24 h with doxycycline in solution or in a hydrogel. The damaged area is where the epithelium meets the stroma. The wound-healing efficacy of doxycycline solution was close to the doxycycline hydrogel for CEES exposed corneas, as the extent of damage was comparatively mild. However, a superior wound healing efficacy was observed with hydrogels over solutions when harshly damaged NM-exposed corneas were treated with doxycycline. CEES: half mustard; NM: mustard; DOXY: doxycycline. (Reprinted with permission from reference [45]. Copyright 2010 Elsevier).

The same research group developed in situ-forming biodegradable hydrogels based on PEG loaded with the bacteriostatic drug doxycycline for the healing of vesicant-induced ocular wounds in rabbit [45]. The hydrogels withstood shear forces and released the cargo (0.25% *w*/*w*) following a bimodal profile with 100% release over one week. The permeation of doxycycline through vesicant wounded corneas was between 2.5- and 3.4-fold higher than in unwounded corneas. Moreover, histology and immunofluorescence studies confirmed a significant decrease of the collagenase matrix metalloproteinase-9 and an improvement of the healing process in vesicant-exposed corneas compared to the free drug delivered in phosphate buffered saline of pH 7.4 (Figure 7).

The regeneration of injured peripheral nerves represents a great challenge for the clinic and it requires the use of scaffolds [46]. Biocompatible hydrogels loaded with drugs could play a fundamental role in this sense because they could be used to accelerate the regenerative process. Guo et al. [47] developed a chitosan conduit containing simvastatin (25 and 50 mg/mL) in Pluronic^®^ F127 hydrogel (25% *w*/*v*) to bind defects in the sciatic nerve of rats. The poloxamer solution is injected into the chitosan conduits and undergoes a phase transition at 37 °C. Preclinical studies in rats showed an improvement in peripheral nerve regeneration with recovery of the functionality. It is important to highlight that injured nerves treated with the drug-loaded hydrogel and the conduit were thicker than controls treated with the hollow duct or with the hydrogel-filled duct without simvastatin, 10 weeks post-surgery. This performance might rely on the increase of the expression of several endogenous neurotrophic factors that has been ascribed to simvastatin.

Regardless of the appeal shown by these systems, it is noteworthy that even though in situ-forming hydrogels can more easily fill well-defined defects and cavities, their free flowing properties may lead to undesired leakage of these precursor solutions into the surrounding tissue or into the bloodstream, unless the defect has well-defined boundaries and the precursor is confined in it until its solidification [40].

#### Smart Hydrogels

The various therapeutic challenges and the improvement of treatment standards led researchers in both academia and industry to devote efforts towards the design and development of “smart” biomaterials in general and hydrogels in particular. The rationale behind them is that they can interplay with the biological environment in a pre-programmed way and thus, display changes in some of their properties (e.g., viscosity) [4] in response to pH, temperature, electric and magnetic fields, among others [44,48,49].

**Figure 8:**
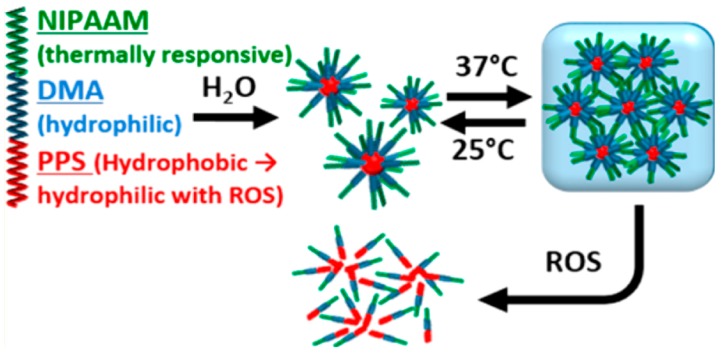
Concept behind hydrogels of poly[(propylenesulfide) (PPS)-(*N*,*N*-dimethylacrylamide) (DMA)-(*N*-isopropylacrylamide) (PNIPAAM) that undergo reversible gelation at 37 °C and degrade upon exposure to ROS. (Reprinted with permission from reference [26]. Copyright 2014 American Chemical Society).

Gupta et al. [26] used the triblock copolymer poly[(propylenesulfide)-(*N*,*N*-dimethylacrylamide)-(*N*-isopropylacrylamide)] to form temperature-sensitive hydrogel endowed with a drug degradation and release mechanism in response to reactive oxygen species (ROS) in vitro (Figure 8). 

When the hydrogel is exposed to ROS, it slowly undergoes a transition to a more hydrophilic poly(propylene sulfide), and finally to a poly(propylsulfone) that triggers the sustained release of drugs “on demand” and subsequent degradation of the hydrogel. In addition, these hydrogels can be used for the encapsulation and release of cells because they possess inherent cellular protection properties and reduce ROS-mediated cell death in vitro [26].

**Figure 9:**
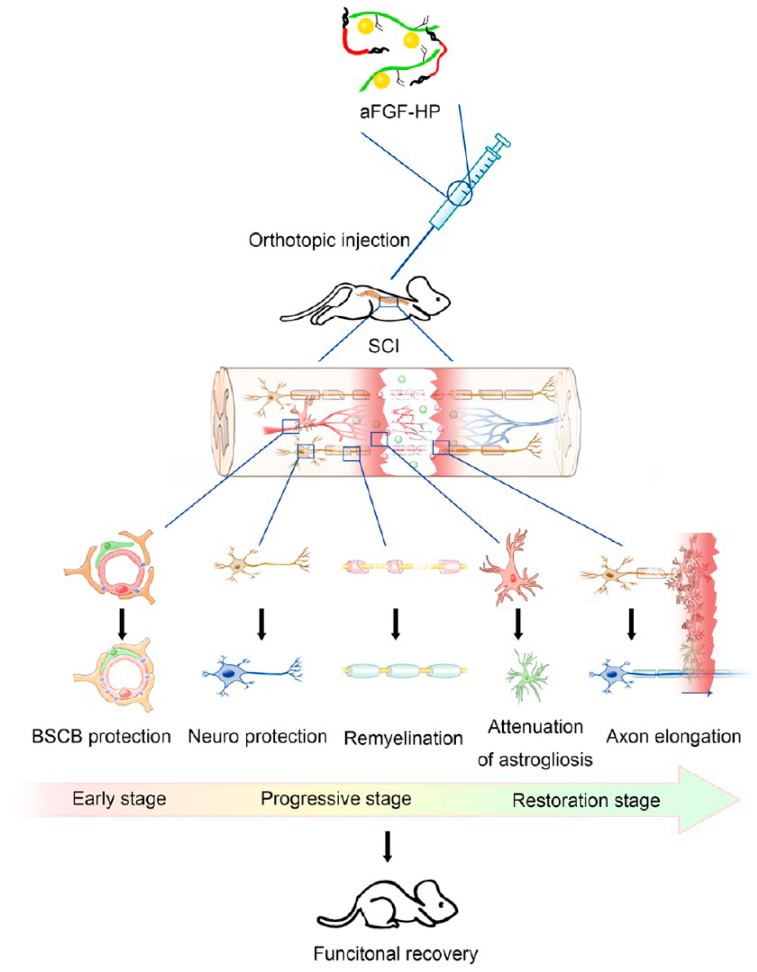
Schematic of aFGF-heparin (HP) thermo-sensitive hydrogels enhance the recovery of spinal cord injury (SCI). The protection of aFGF-HP containing blood-spinal cord barrier (BSCB) protection, neuroprotection, remyelination, attenuation of astrogliosis, axon elongation in three different stages after SCI, which are the main obstacles to recovery of SCI. (Reprinted with permission from reference [50]. Copyright 2017 American Chemical Society).

The synthesis of vehicles that are sensitive to intracellular stimuli is also gaining popularity. These transporters can be created by incorporating various precursors into the hydrogel [15]. Recently, heparin-bound Pluronic^®^ F127 thermo-sensitive hydrogels that load and release acid fibroblast growth factor (aFCF) were used to treat spinal cord injuries (SCIs) [50]. Heparin was added to increase the loading of aFCF, to stabilize its structure, to protect its activity and to control the release. Preclinical studies in rats with SCI showed that animals that were administered the aFCF-loaded heparin-based hydrogel had attenuated disruption of the blood-spinal cord barrier (BSCB), reduction of neuronal apoptosis, production of reactive astrogliosis and increased neuronal and axonal rehabilitation with respect to controls without growth factor or free growth factor administered by the intravenous route (Figure 9) [50].

## 4. Nanogels and Microgels

**Figure 10:**
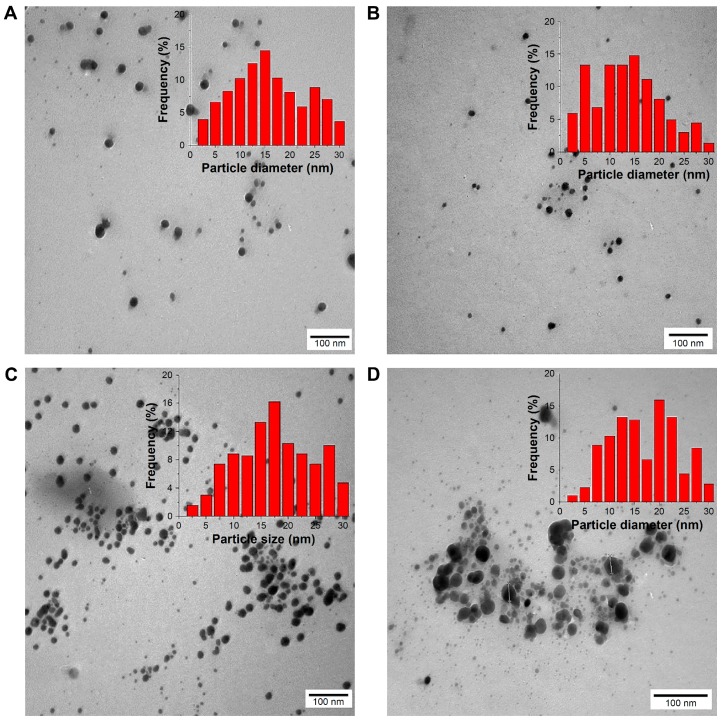
Representative TEM micrographs for the aqueous dried AgNPs (100 μg AgNPs/mL): (**A**) uncoated AgNPs; (**B**) SDS-coated AgNPs; (**C**) PEG-coated AgNPs (×100,000); (**D**) β-CD-coated AgNPs (×140,000) with sizes = 15.7 ± 4.8, 13 ± 4, 19.2 ± 3.6, and 14 ± 4.4 nm, respectively (*n* = 50, bar represents 100 nm). Insets indicate histograms of AgNPs size distribution. Abbreviations: TEM, transmission electron microscopy; AgNPs, silver nanoparticles; SDS, sodium dodecyl sulfate; PEG, polyethylene glycol; β-CD, β-cyclodextrin. (Reprinted from reference [52]).

The development of nanometer-sized hydrogels (nanogels) has attracted the attention of researchers because of their great biomedical potential in drug administration and diagnostics [51]. In addition, this nanotechnology platform can be combined with others to synthesize more complex systems. For example, Mekkawy et al. [52] synthesized silver nanoparticles in the 13–19 nm size range and subsequently coated them with PEG 6000, sodium dodecyl sulfate and β-CD (Figure 10). Then, they studied the antibacterial activity against Gram-positive (e.g., *Staphylococcus aureus*) and Gram-negative (e.g., *Escherichia coli*) bacteria. The values of minimum inhibitory concentration (MIC) and minimum bactericidal concentration (MBC) were in the range of 0.93-7.5 and 3.75-15 μg/mL, respectively, that were lower than those reported in the literature. Then, hydrogels loaded with coated silver nanoparticles were prepared by dispersing the same in gelling agents such as sodium CMC, sodium alginate, HPMC, Pluronic^®^ F127 and chitosan. 

**Figure 11:**
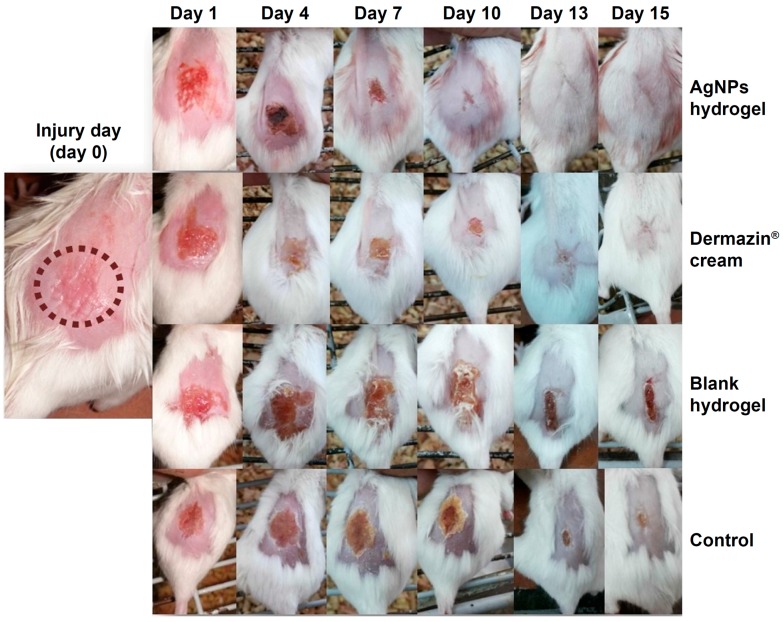
Successive images of representative mice skin abrasion wounds infected with MRSA at different time intervals. Two groups were treated with 0.1% silver nanoparticles (AgNPs) hydrogel and 1% silver sulfadiazine cream. The two other groups were the blank hydrogel-treated group and control untreated mice. Abbreviations: MRSA, methicillin-resistant *Staphylococcus aureus*; AgNPs, silver nanoparticles. (Reprinted from reference [52]).

In vivo results indicated higher antibacterial activity and wound healing capacity of CMC hydrogels loaded with PEG-coated silver nanoparticles with respect to a commercially available sulfadiazine cream (Figure 11) [52].

Another strategy to capitalize on advantageous features of nanotechnology and drug delivery systems at the macro scale is the development of hydrogels formed by self-assembled nanoparticles. In this case, the drug-loaded nanoparticles self-aggregate giving rise to a hierarchical macroscopic hydrogel. After administration, the nanoparticles undergo dissociation and diffuse freely in the physiological milieu, maintaining the favorable characteristics of the nanometric size as a large surface area and easy access to the intracellular space by means of endocytosis. For example, Huang et al. [53] developed nanoparticles made of an (epsilon-caprolactone-co-1,4,8-trioxa[4.6]spiro-9-undecanone)-PEG-poly(epsilon-caprolactone) copolymer loaded with the anti-cancer drug doxorubicin as model drug for peritumoral chemotherapy. In vivo results showed that one single peritumoral injection was more effective in a murine model than multiple intravenous administrations of the free drug and drug-free nanoparticles. This indicated that hydrogels could sustain the release and thus, reduce the administration frequency and minimize systemic toxicity [53].

The combination of microtechnology with hydrogels is also an interesting field for obtaining improved drug delivery systems. Combination strategies may be based on spherical or fiber-like microparticles of hydrogels or microparticles incorporated within macroscopic hydrogels using different techniques. For example, Ahmad et al. [54] synthesized hydrogels of bacterial cellulose graft copolymers with poly(acrylic acid) by electron beam irradiation. The resulting hydrogel sheets were oven dried at 60 °C until constant weight. Then, microparticles were prepared from the hydrogel by grinding and milling the purified sheets. Subsequently, the microparticles were loaded with insulin solution (0.5 mg/mL), filtered and lyophilized. Results showed that the microparticles increased up to 5.9 times the paracellular transport of insulin across the Caco-2/HT29-MTX monolayer model of the intestinal epithelium in vitro when compared to an insulin solution. In addition, insulin-loaded microparticles showed a greater hypoglycemic effect and an increase in relative oral bioavailability of up to 7.45-fold with respect to an insulin solution after oral administration in diabetic rats [54].

## 5. Administration Routes of Hydrogels

Hydrogels have found application in different minimally invasive and invasive administration routes. In the next sections, we will exemplify the great versatility of this technological platform in pharmaceutical development. 

### 5.1. Minimally-Invasive Administration Routes

Buccal administration is becoming a very popular administration route owing to numerous advantages, including the entry of the drug into the systemic circulation and self-administration. However, it requires the appropriate design of the delivery system to maintain its position in the mouth avoiding involuntary ingestion and minimizing the continuous dilution of the drug by the salivary flow. In this sense, hydrogels may be a suitable drug delivery system by this route [55]. For example, Choi et al. [31] developed a thermo-responsive oral mucoadhesive hydrogel for the administration of the anti-cancer drug paclitaxel in the oral mucosa that provides a high local concentration of the drug and it is intended to decrease the systemic side effects normally associated with the intravenous administration. The formulation consisted of a dimethyl-β-CD/paclitaxel inclusion complex which improves drug solubility within a physical hydrogel of Pluronic^®^ F127 with mucoadhesive and sustained release properties. The cytotoxicity of the gels loaded with the inclusion complex was evaluated by the 3-(4,5-dimethylthiazol-2-yl)-2,5-diphenyltetrazolium bromide (MTT) assay using human oral cancer cells (KB cells, a subline of the ubiquitous keratin-forming tumor cell line HeLa). The lowest cell viability was >80% for hydrogels without drug, suggesting that they are not cytotoxic for KB cells. However, the cellular viability of drug hydrogels was considerably lower due to intact paclitaxel cytotoxicity. Since the formulation undergoes gelation at 37 °C, it emerges as a promising approach to treat several oral cancers with reduced systemic toxicity [31].

**Figure 12:**
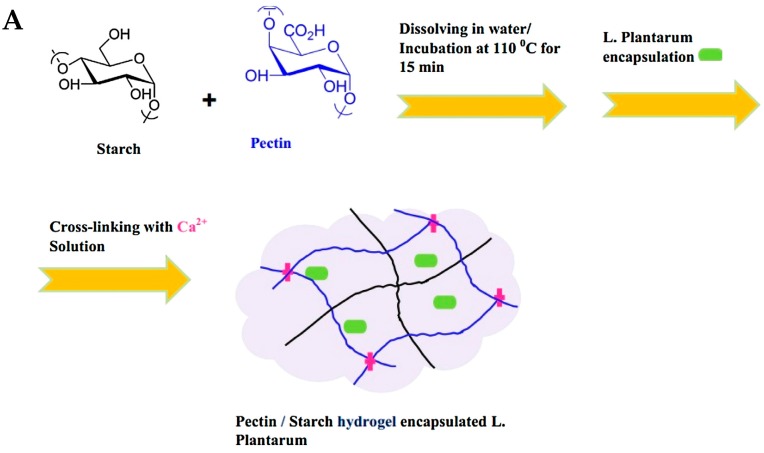

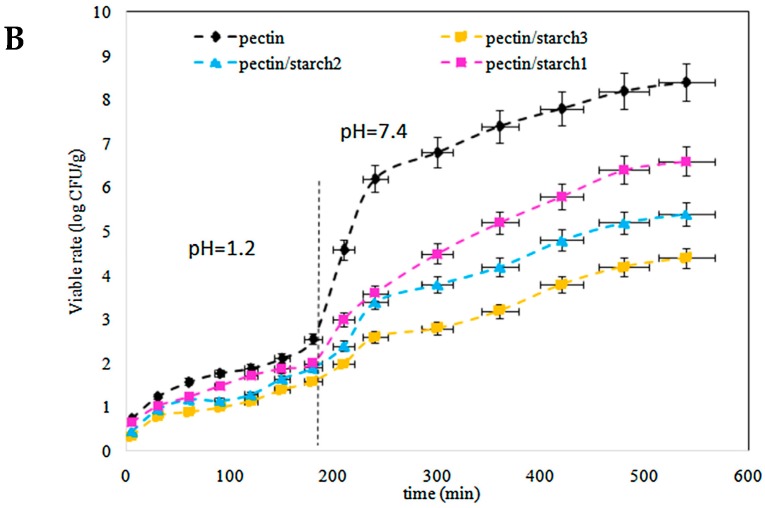
(**A**) Schematic illustration of the preparation of pectin/starch hydrogels encapsulated *Lactobacillus plantarum* (*L. plantarum*) cells. (**B**) Release profile of encapsulated cells in buffered solution with pH 1.2 and pH 7.4; Values shown are means ± standard deviations (*n* = 3). (Reprinted with permission from reference [57]. Copyright 2017 Elsevier).

The oral route is very advantageous over injectable administration due to better patient comfort and compliance and provides a large absorption surface [56]. In this context, Dafe et al. [57] developed a hydrogel made of food grade starch/pectin for the encapsulation and colonic release of the probiotic bacterium *Lactobacillus plantarum* that was encapsulated by the extrusion method. Results demonstrated that the encapsulated cells are resistant to adverse conditions of the simulated gastrointestinal tract and to a bile salt solution as compared to free ones. Then, at more neutral pH conditions that mimic the colon fluids, cells were released and proliferated (Figure 12).

**Figure 13:**
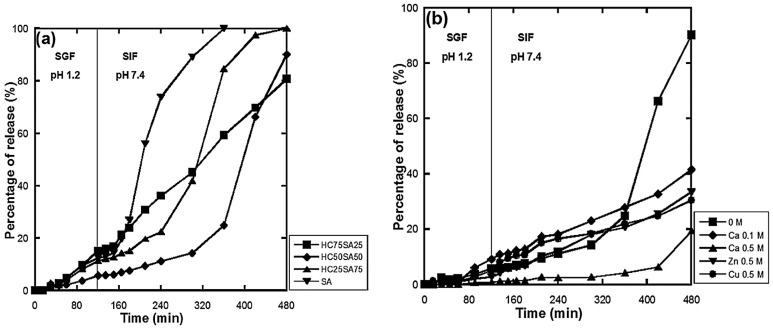
Percentages of paracetamol release from the hydroxyethylacryl chitosan (HC)/sodium alginate (SA) hydrogels after immersing in simulated gastric fluid (SGF) for 2 h followed by simulated intestinal fluid (SIF) for 6 h at 37 °C: (**a**) varying ratios of HC to SA and (**b**) HC50SA50 with varying cross-linker types). (Reprinted with permission from reference [61]. Copyright 2017 Elsevier).

The oral administration of peptides and proteins has become a great challenge in pharmaceutical research [58,59]. In this context, O’Connor et al. [60] developed and characterized systems made of methacrylic acid (MAA), *N*-vinyl pyrrolidone (NVP) and PEG monomethyl ether monomethacrylate (PEGMMA) for the oral delivery of two proteins of different molecular weight, namely insulin and porcine growth hormone. These terpolymer hydrogels are sensitive to different pH conditions. Thus, at pH values <4.8 (e.g., stomach), the carboxylic acid group of MMA is protonated, maintaining the hydrogel in a collapsed state capable of protecting and preventing the release of the encapsulated proteins. As the pH rises above 4.8, as in the small intestine, the carboxylic acid groups are deprotonated, allowing the system to swell and release the therapeutic payload. In addition, these hydrogels exhibit promising mucoadhesive properties for interaction with the lining of the upper small intestine mucosa. Finally, the compatibility of the hydrogels in two model intestinal cell lines (colon adenocarcinoma cells, Caco-2, and mature differentiated goblet cells, HT29-MTX) was investigated and a cytotoxicity of at least 2.5 mg/mL was found [60]. More recently, Treenate and Monvisade [61] developed a pH-sensitive hydrogel of hydroxyethylacryl chitosan and sodium alginate cross-linked with different divalent cationic cross-linkers such as Ca^2+^, Zn^2+^ and Cu^2+^, and used paracetamol as water-soluble model drug. Findings indicated that this combination delayed the degradation time of the hydrogel and that the amount of paracetamol released in the simulated gastric fluid is relatively low (<20%) (Figure 13). Thus, this drug delivery system could be used in site-specific release, such as in small intestine or colon.

Currently, the development of new strategies to increase the residence time of the active substances is a field of constant research in ophthalmic delivery because conventional formulations such as eye drops are easily removed by nasolacrimal drainage and >90% of the drug is lost. Thus, the use of semi-solid formulations that display longer residence time in the corneal surface could be an effective strategy [62]. For example, Huang et al. [63] developed an ophthalmic drug delivery system based on an in situ gelation hydrogel of Pluronic F127^®^ (22% *w*/*v*) and Pluronic^®^ F68 (3.5% *w*/*v*) loaded with betaxolol hydrochloride, a beta blocker used in the treatment of glaucoma. Pharmacokinetic and pharmacodynamic assays in rabbits indicated that the formulation improves the bioavailability and significantly lowers the intraocular pressure with respect to the free drug. Recently, Fabiano et al. [64] developed a thermo-sensitive hydrogel containing mucoadhesive chitosan nanoparticles loaded with 5-fluorouracil for transcorneal administration in the treatment of ocular cancer. The hydrogel could be instilled as drops allowing easy administration and precise dosing. After instillation, the solution gels fast at the temperature of the eye (35 °C). In addition, nanoparticles promoted transcorneal penetration of 5-fluorouracil, at least based on studies in rabbits by aqueous humor analysis. Moreover, the thermo-sensitive hydrogel increased the area-under-the-curve (AUC) between 0 and 8 h, 3.5 times compared to clear eye drops. The maximum drug concentration from the hydrogel with nanoparticles reaches a plateau (0.25–0.3 μg/mL) in a time interval of 0.5 to 7 h, due to the ability of the hydrogel to control the release of the drug following zero-order absorption kinetics [64].

The nasal mucosa has become an appealing administration route not only for local treatments (e.g., allergies) but also for systemic delivery because it is self-administered, it does not require sterility and it is virtually painless. From a pharmacokinetic point of view, absorption is rapid due to the existence of a rich vascularization and a highly permeable structure within the nasal membranes and it also surpasses hepatic first-pass metabolism [65]. An additional advantage is that depending on the administration site in the nose, drugs could follow the nose-to-brain pathway, a transport that is favored for nanoparticles and that overcomes the presence blood-brain barrier that prevents the arrival of many drugs from the systemic circulation [66]. In this framework, Khan et al. [67] developed in situ gelling formulations using chitosan and hydroxypropyl methylcellulose (HPMC) for the intranasal administration of ropinirole, a drug used in the treatment of Parkinson’s disease, with the objective of its direct arrival in the brain. In vivo bioavailability and brain uptake assays were performed in albino rats following intranasal gel administration with ropinirole-^99m^Tc, an intranasal ropinirole solution and an intravenous ropinirole solution. The absolute bioavailability of the drug from the in situ gel formulation was 82%. The AUC value in the brain after administration of the gel in situ between 0 and 480 min was 8.5 times greater than that obtained intravenously and 3 times higher than that obtained with the solution of the intranasal drug. This confirmed that the nose-brain transport of the gel formulation in situ with ropinirole could represent a breakthrough in the treatment of Parkinson’s disease [67]. However, it is important to note that the site of administration within the nasal cavity should be appropriate to favor passage into the central nervous system and reduce passage through systemic pathways. On the other hand, Mahajan and Gattani [68] obtained in situ gels of metoclopramide hydrochloride, a potent antiemetic, for intranasal administration. The gels were prepared using different ratios of gellan gum, carbopol 934 P and mannitol. In vitro drug permeation studies across the nasal sheep mucosa showed that the effective passage way could be increased using a formulation with 0.15% *w*/*v* carbopol or more. In addition, histological examination confirmed no mucosal damage during in vitro permeability studies. Finally, the bioavailability assay of the drug in rabbits showed a significant increase from 40.7% in the case of the drug in solution to 54.6% in the case of the gel [68].

The skin is an attractive and accessible route for the systemic administration of drugs from matrices such as those formed by hydrogels, but the presence of the stratum corneum should be surpassed to ensure efficient absorption. For example, Arafa and Ayoub [69] designed a hydrogel of HPMC and carbopol 934 loaded with cholesterol niosomes and Span^®^ 60 loaded with pregabalin to prevent or diminish the adverse effects produced by the drug after oral administration. Pregabalin is a drug used in fibromyalgia syndrome and acute pains such as sciatic neuralgia. However, it produces many adverse effects such as dizziness, drowsiness, dry mouth, blurred vision, difficulty concentrating, hypersensitivity and decreased platelet count. The parameters of the hydrogel were optimized using an experimental design. In vitro release assays evidenced a pattern of controlled release. In addition, the study of permeation in excised skin of rats using Franz diffusion cells showed that the passage of drug-loaded niosomes was significantly greater than the conventional pregabalin hydrogels [69]. 

The vaginal route is an excellent route for both systemic and local applications since gastrointestinal fluids and first-pass liver metabolism are bypassed. In addition, dense vascularization and high mucosal permeability make this pathway widely used for the administration of many drugs such as hormones, peptides, antimicrobials and antifungals, among others [70]. In this framework, Malli et al. [71] designed a thermo-sensitive and mucoadhesive hydrogel of Pluronic^®^ F127 and chitosan containing metronidazole for topical application to the vaginal mucosa to treat infections by the protozoan microorganism *Trichomonas vaginalis*. This topical administration system could reduce the adverse effects produced by the drug in standard oral therapy because as it limits the absorption through the vaginal mucosa, while preserving its antiparasitic activity. In addition, the mucoadhesive properties of the hydrogel controlled the release over time ensuring a more prolonged activity and gelation in situ facilitates self-administration of a liquid capable of achieving better coverage of the vaginal mucosa that becomes semisolid at body temperature. The accumulated absorption of the drug through ex vivo pig vaginal mucosa mounted on a Franz diffusion cell presented a reduction with respect to the drug in solution and thus, it is expected to result in less systemic adverse effects. Finally, the activity of metronidazole formulated in the hydrogel was preserved since the viability curve evaluated in vitro was similar to the free counterpart [71]. Vaginal microbicidal hydrogels could be a barrier against viral infections and prevent the spreading of the virus through the vaginal mucosa. In this sense, Bouchemal et al. [72] developed thermo-sensitive hydrogels of Pluronic^®^ F127 (20% *w*/*w*) and HPMC (1% *w*/*w*) containing carboxyl groups-modified polystyrene particles to mimic the size and surface charge of Type 1 human immunodeficiency virus (HIV-1). Analysis of the trajectories of the fluorescently labeled particles showed that the mobility was decreased by the hydrogels compared to cervico-vaginal mucus of traditional hydroxyethyl cellulose hydrogels used as negative controls. In addition, the incorporation of the CD4 M48U1 mini-peptide used as an anti-HIV-1 molecule in a mixture of the polymers did not affect its anti-HIV-1 activity as compared to the conventional hydrogel. Therefore, this type of formulation could be used as a topical microbicide containing an anti-HIV compound, as it would simultaneously act as a physical and pharmacological barrier [72].

Another administration route gaining interest is the rectal one because it can reduce the adverse effects of orally administered drugs, improve patient compliance with respect to injections and be used in special conditions such as unconscious patients, with difficulty of swallowing or under chronic therapies where other routes are not possible. It can also be used in local treatments. For example, Cole et al. [73] developed a 4000 g/mol PEG-based hydrogel in the form of morphine-containing suppositories for sustained release of the drug following rectal administration. Pharmacokinetic results in 5 healthy volunteers showed that the drug was released at a constant rate for at least 12 h. This could improve the pharmacotherapy of patients with chronic pain.

### 5.2. Invasive Administration Routes

Injectable drug administration involves intravenous, intramuscular, intradermal and subcutaneous, among others. It may also include intra- or peritumoral administration when the drug is delivered directly into the tumor or at a site close to it. The drug carriers could be aqueous or oily according to the route selected. Hereinafter, we will give some examples of drug delivery via injection of hydrogels.

Chen et al. [30] developed an injectable thermo-responsive hydrogel with mechanical stability and biocompatibility as a controlled drug delivery vehicle for cancer therapy. The matrix used was Pluronic^®^ F127 that incorporated hexamethylene diisocyanate links to increase the mechanical stability. The hydrogel structure was maintained for 30 days ensuring sustained release of the incorporated drug over an extended period. This copolymer was then added to hyaluronic acid, a natural biocompatible polymer, to obtain the final nanocomposite hydrogel system that can self-assemble spontaneously into a micellar structure with a size of 100 to 200 nm. In vitro studies evidenced that the incorporated drug, doxorubicin, was released for more than 28 days. By using this strategy, the viability of tumor cells (human breast cancer cells, MCF-7) and tumor size in mice was significantly reduced with the incubation time [30]. Seib et al. [74] developed self-assembling silk protein hydrogels also loaded with doxorubicin for the treatment of focal breast cancer and compared this system with the drug administered intravenously. These hydrogels released the drug for 4 weeks in amounts that could be precisely adjusted by varying the silk content in the matrix. In addition, its use improved both the safety and efficacy of the drug. First, the cytotoxicity of doxorubicin-loaded and doxorubicin-free hydrogels was evaluated using MDA-MB-231 and MCF-7 (human) breast cancer cell lines and 3-day exposures. The viability of MCF-7 was reduced to 10% after exposure to the free drug and to 20% for silk hydrogels. For MDA-MB-231 cells, viability was reduced to 23% for all treatment groups. In addition, a long-term test was performed for 12 days. Control cultures of MDA-MB-231 and MCF-7 showed exponential cell growth at days 2 to 6 and lower growth over the remaining days. In contrast, doxorubicin-treated groups showed no or minimal growth during the first 6 days in the culture. However, they exhibited significant growth over the rest of the study. In contrast, doxorubicin-loaded hydrogels inhibited cell growth. Regarding efficacy, doxorubicin-loaded hydrogels administered by bilateral injections near the tumor site showed better antitumor activity than the equivalent amount of drug administered intravenously. In addition to reducing the primary tumor in mice with human breast cancer tumor cell xenografts, hydrogels reduced metastasis and were well tolerated. Thus, two of the five animals treated with doxorubicin-loaded silk hydrogels presented complete tumor regression, determined by bioluminescence and necropsy. Therefore, this system could be suitable for local chemotherapy administration and promising in breast cancer therapy to improve loco-regional control of breast cancer [74]. More recently, Wu et al. [75] developed a thermo-sensitive hydrogel of cross-linked nanoparticles of poly(vinyl caprolactam)-poly(vinyl acetate)-PEG (known as Soluplus^®^) and tacrolimus for local therapy of rheumatoid arthritis. The therapeutic efficacy in rats with adjuvant-induced arthritis significantly increased from day 10 to 17 after a single dose of FK-506 loaded in 10% and 20% Soluplus^®^ hydrogels with respect to uncharged hydrogels owing to sustained drug release and prolonged retention at the injection site. 

## 6. Conclusions and Future Directions

The physical and mechanical properties of hydrogels, together with their biocompatibility and biodegradability characteristics, have made these materials attractive to a wide range of technological applications. Adjustment of the hydrogel preparation conditions by varying the different parameters allows designing suitable systems for potential applications in the fields of drug delivery, tissue engineering and 3D cell culture and has found its space in the market. The use of “smart” polymers capable of responding to various stimuli such as changes in pH, temperature, ionic strength or enzymatic degradation allows administration of polymeric liquids that undergo gelation under physiological conditions leading to the formation of hydrogels in situ. However, preformed hydrogels are useful in various applications as wound scaffolds while maintaining the characteristics of transparency allowing inspection of wounds, as well as the ability to release antimicrobial or anti-inflammatory drugs and growth factors from their structure by aiding the regeneration of the tissue. They also allow administration by injection of the solid hydrogel capable of “thinning” during its administration and subsequent recovery of the mechanical and morphological properties of the original solid. In addition, hydrogels can be functionalized with a radiopaque that provides X-ray opacity and allows them to be used as biomedical implants for in vivo visualization and evaluation of the ability of the hydrogel to prevent postoperative adhesions [76]. As for the administration routes, hydrogels are very versatile allowing oral, injectable, dermal, vaginal, ophthalmic, and nasal delivery. In summary, hydrogels represent one of the most versatile technological platforms for pharmaceutical innovation. On the other hand, the successful bench-to-bedside translation remains elusive and it is not in accordance with the vast scientific literature probably due to the challenges faced for the establishment of standardized, scalable and economically viable production processes. This appears as a critical stage of development that is neglected in most scientific works. Only then, the true potential of hydrogels could impact the quality of the treatments available for the benefit of patients.

## References

[1] Anumolu S.S., Singh Y., Gao D., Stein S., Sinko P.J. (2009). Design and evaluation of novel fast forming pilocarpine-loaded ocular hydrogels for sustained pharmacological response. J. Control Release.

[2] Gaharwar A.K., Peppas N.A., Khademhosseini A. (2014). Nanocomposite hydrogels for biomedical applications. Biotechnol. Bioeng..

[3] Onuki Y., Hasegawa N., Kida C., Obata Y., Takayama K. (2014). Study of the contribution of the state of water to the gel properties of a photocrosslinked polyacrylic acid hydrogel using magnetic resonance imaging. J. Pharm. Sci..

[4] Rossi B., Venuti V., D’Amico F., Gessini A., Mele A., Punta C., Melone L., Crupi V., Majolino D., Masciovecchio C. (2016). Guest-matrix interactions affect the solvation of cyclodextrin-based polymeric hydrogels: A UV Raman scattering study. Soft Matter.

[5] Schoener C.A., Hutson H.N., Peppas N.A. (2013). pH-Responsive Hydrogels with Dispersed Hydrophobic Nanoparticles for the Oral Delivery of Chemotherapeutics. J. Biomed. Mater. Res. A.

[6] Hou K., Wang H., Lin Y., Chen S., Yang S., Cheng Y., Hsiao B.S., Zhu M. (2016). Large Scale Production of Continuous Hydrogel Fibers with Anisotropic Swelling Behavior by Dynamic-Crosslinking-Spinning. Macromol. Rapid Commun..

[7] Concheiro A., Alvarez-Lorenzo C. (2013). Chemically cross-linked and grafted cyclodextrin hydrogels: From nanostructures to drug-eluting medical devices. Adv. Drug Deliv. Rev..

[8] Ye X., Yin H., Lu Y., Zhang H., Wang H. (2016). Evaluation of Hydrogel Suppositories for Delivery of 5-Aminolevulinic Acid and Hematoporphyrin Monomethyl Ether to Rectal Tumors. Molecules.

[9] Siepmann J., Peppas N.A. (2011). Higuchi equation: Derivation, applications, use and misuse. Int. J. Pharm..

[10] Peppas N.A., Moynihan H.J. (1985). Solute diffusion in swollen membranes. IV. Theories for moderately swollen networks. J. Appl. Polym. Sci..

[11] Harland R.S., Peppas N.A. (1989). Solute diffusion in swollen membranes. VII. Diffusion in semicrystalline networks. Colloid Polym. Sci..

[12] Caldorera-Moore A., Peppas N.A. (2009). Micro- and Nanotechnologies for Intelligent and Responsive Biomaterial-Based Medical Systems. Adv. Drug Deliv. Rev..

[13] Singh A., Peppas N.A. (2014). Hydrogels and Scaffolds for Immunomodulation. Adv. Mater..

[14] Elviri L., Bianchera A., Bergonzi C., Bettini R. (2016). Controlled local drug delivery strategies from chitosan hydrogels for wound healing. Expert Opin. Drug Deliv..

[15] Pérez E., Fernández A., Olmo R., Teijón J.M., Blanco M.D. (2014). pH and glutathion-responsive hydrogel for localized delivery of paclitaxel. Colloids Surf. B Biointerfaces.

[16] Silva R., Fabry B., Boccaccini A.R. (2014). Fibrous protein-based hydrogels for cell encapsulation. Biomaterials.

[17] Zhang H., Wang L., Song L., Niu G., Cao H., Wang G., Yang H., Zhu S. (2010). Controllable properties and microstructure of hydrogels based on crosslinked poly(ethylene glycol) diacrylates with different molecular weights. J. Appl. Polym. Sci..

[18] Slaughter B.V., Khurshid S.S., Fisher O.Z., Khademhosseini A., Peppas N.A. (2009). Hydrogels in Regenerative Medicine. Adv. Mater..

[19] Naseri N., Deepa B., Mathew A.P., Oksman K., Girandon L. (2016). Nanocellulose-Based Interpenetrating Polymer Network (IPN) Hydrogels for Cartilage Applications. Biomacromolecules.

[20] Straccia M.C., d’Ayala G.G., Romano I., Oliva A., Laurienzo P. (2015). Alginate hydrogels coated with chitosan for wound dressing. Mar. Drugs.

[21] Alvarez-Lorenzo C., dos Santos F.R., Sosnik A., Torres-Labandeira J., Concheiro A., Stein D.B. (2009). Cyclodextrin-based hydrogels as highly versatile drug delivery systems. Handbook of Hydrogels: Properties, Preparation and Applications.

[22] Machín R., Isasi J.R., Vélaz I. (2012). β-Cyclodextrin hydrogels as potential drug delivery systems. Carbohydr. Polym..

[23] Mennini N., Casella G., Cirri M., Maestrelli F., Mura P. (2016). Development of cyclodextrin hydrogels for vaginal delivery of dehydroepiandrosterone. J. Pharm. Pharmacol..

[24] Sosnik A. (2007). Design of Injectable biomaterials for biomedical and pharmaceutical applications: Past, present and future of the in situ generated implants. ARS Pharm..

[25] Tsitsilianis C. (2010). Responsive reversible hydrogels from associative “smart” macromolecules. Soft Matter.

[26] Gupta M.K., Martin J.R., Werfel T.A., Shen T., Page J.M., Duvall C.L. (2014). Cell protective, ABC triblock polymer-based thermoresponsive hydrogels with ROS-triggered degradation and drug release. J. Am. Chem. Soc..

[27] Marchesan S., Qu Y., Waddington L.J., Easton C.D., Glattauer V., Lithgow T.J., McLean K.M., Forsythe J.S., Hartley P.G. (2013). Self-assembly of ciprofloxacin and a tripeptide into an antimicrobial nanostructured hydrogel. Biomaterials.

[28] Slaughter B.V., Blanchard A.T., Maass K.F., Peppas N.A. (2015). Dynamic swelling behavior of interpenetrating polymer networks in response to temperature and pH. J. Appl. Polym. Sci..

[29] Chiappetta D.A., Sosnik A. (2007). Poly(ethylene oxide)-poly(propylene oxide) block copolymer micelles as drug delivery agents: Improved hydrosolubility, stability and bioavailability of drugs. Eur. J. Pharm. Biopharm..

[30] Chen Y.Y., Wu H.C., Sun J.S., Dong G.C., Wang T.W. (2013). Injectable and thermoresponsive self-assembled nanocomposite hydrogel for long-term anticancer drug delivery. Langmuir.

[31] Choi S.G., Lee S.E., Kang B.S., Ng C.L., Davaa E., Park J.S. (2014). Thermosensitive and mucoadhesive sol-gel composites of paclitaxel/dimethyl-β-cyclodextrin for buccal delivery. PLoS ONE.

[32] Jiang J., Burger C., Li C., Li J., Lin M.Y., Colby R.H., Rafailovich M.H., Sokolov J.C. (2007). Shear-Induced Layered Structure of Polymeric Micelles by SANS. Macromolecules.

[33] Varshosaz J., Tabbakhian M., Salmani Z. (2008). Designing of a Thermosensitive Chitosan/Poloxamer In Situ Gel for Ocular Delivery of Ciprofloxacin. Open Drug Deliv. J..

[34] Esposito E., Carotta V., Scabbia A., Trombelli L., D’Antona P., Menegatti E., Nastruzzi C. (1996). Comparative analysis of tetracycline-containing dental gels: Poloxamer- and monoglyceride-based formulations. Int. J. Pharm..

[35] Sosnik A., Cohn D. (2003). Poly(ethylene glycol)-poly(epsilon-caprolactone) block oligomers as injectable materials. Polymer.

[36] Sosnik A., Cohn D., San Román J., Abraham G.A. (2003). Crosslinkable PEO-PPO-PEO-based reverse thermo-responsive gels as potentially injectable materials. J. Biomater. Sci. Polym. Ed..

[37] Cohn D., Sosnik A., Levy A. (2003). Improved reverse thermo-responsive polymeric systems. Biomaterials.

[38] Kumar S., Himmelstein K.J. (1995). Modification of in situ gelling behavior of carbopol solutions by hydroxypropyl methylcellulose. J. Pharm. Sci..

[39] Huang Y.C., Huang K.Y., Yang B.Y., Ko C.H., Huang H.M. (2016). Fabrication of Novel Hydrogel with Berberine-Enriched Carboxymethylcellulose and Hyaluronic Acid as an Anti-Inflammatory Barrier Membrane. Biomed Res. Int..

[40] Yan C., Altunbas A., Yucel T., Nagarkar R.P., Schneider J.P., Pochan D.J. (2010). Injectable solid hydrogel: Mechanism of shear-thinning and immediate recovery of injectable β-hairpin peptide hydrogels. Soft Matter.

[41] Riber L., Burmølle M., Alm M., Milani S.M., Thomsen P., Hansen L.H., Sørensen S.J. (2016). Enhanced plasmid loss in bacterial populations exposed to the antimicrobial compound irgasan delivered from interpenetrating polymer network silicone hydrogels. Plasmid.

[42] Tummala G.K., Rojas R., Mihranyan A. (2016). Poly(vinyl alcohol) Hydrogels Reinforced with Nanocellulose for Ophthalmic Applications: General Characteristics and Optical Properties. J. Phys. Chem. B.

[43] Dos Santos J.F., Alvarez-Lorenzo C., Silva M., Balsa L., Couceiro J., Torres-Labandeira J.J., Concheiro A. (2009). Soft contact lenses functionalized with pendant cyclodextrins for controlled drug delivery. Biomaterials.

[44] Chassenieux C., Tsitsilianis C. (2016). Recent trends in pH/thermo-responsive self-assembling hydrogels: From polyions to peptide-based polymeric gelators. Soft Matter.

[45] Anumolu S.S., DeSantis A.S., Menjoge A.R., Hahn R.A., Beloni J.A., Gordon M.K., Sinko P.J. (2010). Doxycycline loaded poly(ethylene glycol) hydrogels for healing vesicant-induced ocular wounds. Biomaterials.

[46] Luo Y., Shoichet M.S. (2004). A photolabile hydrogel for guided three-dimensional cell growth and migration. Nat. Mater..

[47] Guo Q., Liu C., Hai B., Ma T., Zhang W., Tan J., Fu X., Wang H., Xu Y., Song C. (2017). Chitosan conduits filled with simvastatin/Pluronic F-127 hydrogel promote peripheral nerve regeneration in rats. J. Biomed. Mater. Res. B Appl. Biomater..

[48] Osman A., Oner E.T., Eroglu M.S. (2017). Novel levan and pNIPA temperature sensitive hydrogels for 5-ASA controlled release. Carbohydr. Polym..

[49] Cho S.H., Kim A., Shin W., Heo M.B., Noh H.J., Hong K.S., Cho J.H., Lim Y.T. (2017). Photothermal-modulated drug delivery and magnetic relaxation based on collagen/poly(γ-glutamic acid) hydrogel. Int. J. Nanomed..

[50] Wang Q., He Y., Zhao Y., Xie H., Lin Q., He Z., Wang X., Li J., Zhang H., Wang C. (2017). A Thermosensitive Heparin-Poloxamer Hydrogel Bridges aFGF to Treat Spinal Cord Injury. ACS Appl. Mater. Interfaces.

[51] Marek S.R., Conn C.A., Peppas N.A. (2010). Cationic Nanogels Based On Diethylaminoethyl Methacrylate. Polymer.

[52] Mekkawy A.I., El-Mokhtar M.A., Nafady N.A., Yousef N., Hamad M.A., El-Shanawany S.M., Ibrahim E.H., Elsabahy M. (2017). In vitro and in vivo evaluation of biologically synthesized silver nanoparticles for topical applications: Effect of surface coating and loading into hydrogels. Int. J. Nanomed..

[53] Huang P., Song H., Zhang Y., Liu J., Zhang J., Wang W., Liu J., Li C., Kong D. (2016). Bridging the Gap between Macroscale Drug Delivery Systems and Nanomedicines: A Nanoparticle-Assembled Thermosensitive Hydrogel for Peritumoral Chemotherapy. ACS Appl. Mater. Interfaces.

[54] Ahmad N., Mohd Amin M.C., Ismail I., Buang F. (2016). Enhancement of oral insulin bioavailability: In vitro and in vivo assessment of nanoporous stimuli-responsive hydrogel microparticles. Expert Opin. Drug Deliv..

[55] Kamel R., Mahmoud A., El-Feky G. (2012). Double-phase hydrogel for buccal delivery of tramadol. Drug Dev. Ind. Pharm..

[56] Forbes D.C., Peppas N.A. (2012). Oral delivery of small RNA and DNA. J. Control. Release.

[57] Dafe A., Etemadi H., Dilmaghani A., Mahdavinia G.R. (2017). Investigation of pectin/starch hydrogel as a carrier for oral delivery of probiotic bacteria. Int. J. Biol. Macromol..

[58] Alonso-Sande M., Csaba N.S., Alonso M.J., Russell-Jones G. (2016). New perspectives in oral peptide and protein delivery: From nanocarrier design to in vivo effectiveness. Advances and Challenges Oral Delivery of Macromolecules.

[59] Brayden D.J., Alonso M.J. (2016). Oral delivery of peptides: Opportunities and issues for translation. Adv. Drug Deliv. Rev..

[60] O’Connor C., Steichen S., Peppas N.A. (2017). Development and characterization of stimuli-responsive hydrogel microcarriers for oral protein delivery. J. Biomed. Mater. Res. A.

[61] Treenate P., Monvisade P. (2017). In vitro drug release profiles of pH-sensitive hydroxyethylacryl chitosan/sodium alginate hydrogels using paracetamol as a soluble model drug. Int. J. Biol. Macromol..

[62] Fernández-Ferreiro A., Silva-Rodriguez J., Otero-Espinar F.J., González-Barcia M., Lamas M.J., Ruibal A., Luaces-Rodríguez A., Vieites-Prado A., Lema I., Herranz M. (2017). In vivo eye surface residence determination by high-resolution scintigraphy of a novel ion-sensitive hydrogel based on gellan gum and kappa-carrageenan. Eur. J. Pharm. Biopharm..

[63] Huang W., Zhang N., Hua H., Liu T., Tang Y., Fu L., Yang Y., Ma X., Zhao Y. (2016). Preparation, pharmacokinetics and pharmacodynamics of ophthalmic thermosensitive in situ hydrogel of betaxolol hydrochloride. Biomed. Pharmacother..

[64] Fabiano A., Bizzarri R., Zambito Y. (2017). Thermosensitive hydrogel based on chitosan and its derivatives containing medicated nanoparticles for transcorneal administration of 5-fluorouracil. Int. J. Nanomed..

[65] Zaki N.M., Awad G.A., Mortada N.D., Abd El Hady S.S. (2007). Enhanced bioavailability of metoclopramide HCl by intranasal administration of a mucoadhesive in situ gel with modulated rheological and mucociliary transport properties. Eur. J. Pharm. Sci..

[66] Mistry A., Stolnik S., Illum L. (2009). Nanoparticles for direct nose-to-brain delivery of drugs. Int. J. Pharm..

[67] Khan S., Patil K., Bobade N., Yeole P., Gaikwad R. (2010). Formulation of intranasal mucoadhesive temperature-mediated in situ gel containing ropinirole and evaluation of brain targeting efficiency in rats. J. Drug Target..

[68] Mahajan H.S., Gattani S. (2010). In situ gels of Metoclopramide Hydrochloride for intranasal delivery: In vitro evaluation and in vivo pharmacokinetic study in rabbits. Drug Deliv..

[69] Arafa M.G., Ayoub B.M. (2017). DOE Optimization of Nano-based Carrier of Pregabalin as Hydrogel: New Therapeutic & Chemometric Approaches for Controlled Drug Delivery Systems. Sci. Rep..

[70] Furst T., Piette M., Lechanteur A., Evrard B., Piel G. (2015). Mucoadhesive cellulosic derivative sponges as drug delivery system for vaginal application. Eur. J. Pharm. Biopharm..

[71] Malli S., Bories C., Pradines B., Loiseau P.M., Ponchel G., Bouchemal K. (2017). In situ forming pluronic^®^ F127/chitosan hydrogel limits metronidazole transmucosal absorption. Eur. J. Pharm. Biopharm..

[72] Bouchemal K., Aka-Any-Grah A., Dereuddre-Bosquet N., Martin L., Lievin-Le-Moal V., Le Grand R., Nicolas V., Gibellini D., Lembo D., Poüs C. (2015). Thermosensitive and mucoadhesive pluronic-hydroxypropylmethylcellulose hydrogel containing the mini-CD4 M48U1 is a promising efficient barrier against HIV diffusion through macaque cervicovaginal mucus. Antimicrob. Agents Chemother..

[73] Cole L., Hanning C.D., Robertson S., Quinn K. (1990). Further development of a morphine hydrogel suppository. Br. J. Clin. Pharmacol..

[74] Seib F.P., Pritchard E.M., Kaplan D.L. (2013). Self-assembling doxorubicin silk hydrogels for the focal treatment of primary breast cancer. Adv. Funct. Mater..

[75] Wu H., Wang K., Wang H., Chen F., Huang W., Chen Y., Chen J., Tao J., Wen X., Xiong S. (2017). Novel self-assembled tacrolimus nanoparticles cross-linking thermosensitive hydrogels for local rheumatoid arthritis therapy. Colloids Surf. B Biointerfaces.

[76] Lei K., Chen Y., Wang J., Peng X., Yu L., Ding J. (2017). Non-invasive Monitoring of In Vivo Degradation of a Radiopaque Thermoreversible Hydrogel and Its Efficacy in Preventing Post-operative Adhesions. Acta Biomater..

